# Data on retinoic acid and reduced serum concentration induced differentiation of Neuro-2a neuroblastoma cells

**DOI:** 10.1016/j.dib.2018.11.097

**Published:** 2018-11-23

**Authors:** Mukesh Kumar, Anju Katyal

**Affiliations:** Dr. B. R. Ambedkar Center for Biomedical Research, University of Delhi, 110007, India

**Keywords:** Neuro-2a, Retinoic acid, Reduced serum

## Abstract

The present data describe the relative neuro-2a cellular differentiation induced by reducing serum concentration (0.1% FBS) in DMEM in the presence/absence of 20 μM retinoic acid (RA). Neurite outgrowth was observed within 24 h in DMEM supplemented with reduced serum and retinoic acid (GpIV). The CFSE based proliferation assay data signified cessation of neuro-2a cellular proliferation in GpIV. An increase in the number of cells arrested at G_0_/G_1_ phase was also evident in GpIV and DMEM supplemented with 0.1% FBS (GpIII). Moreover, GpIV cells had improved mRNA and protein expression of Rbfox3/NeuN and choline acetyltransferase (ChAT).

## Specifications table

**Table:** 

Subject area	Biology
More specific subject area	Neuroscience.
Type of data	Image, graph, figure, histogram.
How data was acquired	Microscope, Chemiluminescence, BD FACSCalibur™, Biorad CFX96 Real-Time PCR.
Data format	Analyzed.
Experimental factors	Retinoic acid (20 μM), FBS, DMEM, time (24 h)
Experimental features	Neuro-2a cells were incubated in the differentiation media consisting of retinoic Acid (20 μM), 0.1% FBS, DMEM for a duration of 24 h.
Data source location	New Delhi, India.
Data accessibility	Data is with this article only.

## Value of the data

• The present data describe the cell culture conditions, driving reproducible neuro-2a cellular differentiation within 24 h and will be useful for people working in this domain.
• The data describe a stepwise strategy to be used for characterizing neuro-2a cellular differentiation.
• The present data demonstrated induced expression of ChAT that can be helpful for researchers in their experimental planning.

## 1 Data

**Fig. 1:**
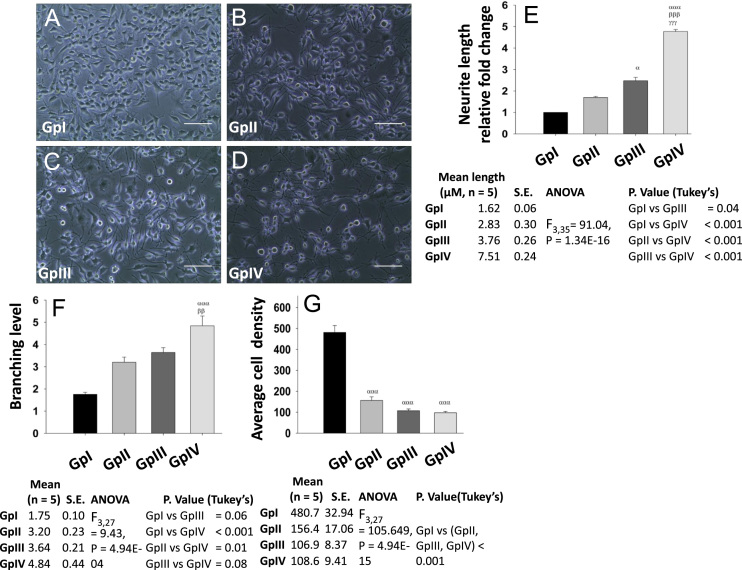
Phase contrast microscopy for morphological analysis. Neuro-2a cells were subjected to various culture conditions, namely DMEM + 10% FBS (GpI), DMEM + 10% FBS + 20 μM Retinoic acid (GpII), DMEM + 0.1% FBS (GpIII), DMEM + 0.1% FBS + 20 μM Retinoic acid (GpIV) and then photographed with Nikon-C100 at 20× magnification. Scale bar 5 μM. Data are represented as photomicrographs (A–D) and its analysis of neurite length (E), the branching level (F) and average cell density (cells/475 μm^2^, G) in bar graphs. Values are mean ± S.E. *P* ≤ 0.05 (*α*, *β*, *γ*), *P* ≤ 0.01 (*αα*, *ββ*, *γγ*), *P* ≤ 0.001 (*ααα*, *βββ*, *γγγ*) were considered to be statistically significant. *α*, Compared to GpI; *β*, Compared to GpII; *γ*, Compared to GpIII. Additionally, mean values, S.E., ANOVA, and Tukey׳s multiple comparisons statistics (post hoc) are shown in tabular form below their respective graphs.

**Fig. 2:**
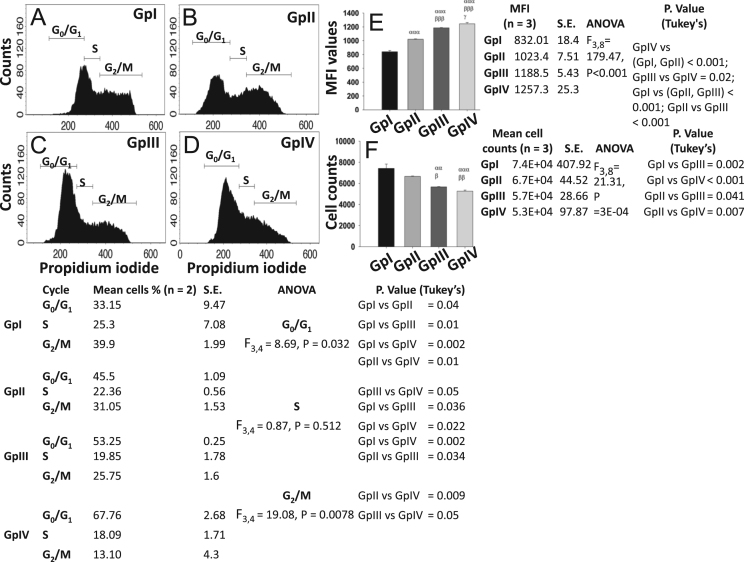
Modulation in cell cycle and proliferation pattern of neuro-2a under varied serum conditions with presence/absence of retinoic acid. Neuro-2a cells were subjected to culture conditions, namely DMEM + 10% FBS (GpI), DMEM + 10% FBS + 20 μM Retinoic acid (GpII), DMEM + O.1% FBS (GpIII), DMEM + 0.1% FBS + 20 μM Retinoic acid (GpIV) for 24 h, processed as per respective protocols and then scanned using FACS Calibur™. Cell cycle data are represented as the histogram (A–D) and CFSE assay data is represented in bar graphs (E, F) showing mean fluorescence intensity values (MFI values) and cell counts (cells/9.6 cm^2^) respectively. Values are mean ± S.E. *P* ≤ 0.05 (*α*, *β*, *γ*), P ≤ 0.01 (αα, ββ, γγ), *P* ≤ 0.001 (*ααα*, *βββ*, *γγγ*) were considered to be statistically significant. *α*, Compared to GpI; *β*, Compared to GpII; *γ*, Compared to GpIII. Additionally, mean data values, S.E., ANOVA statistics and *P*. Values from Tukey׳s multiple comparisons (post hoc) are represented besides respective graphical representations.

**Fig. 3:**
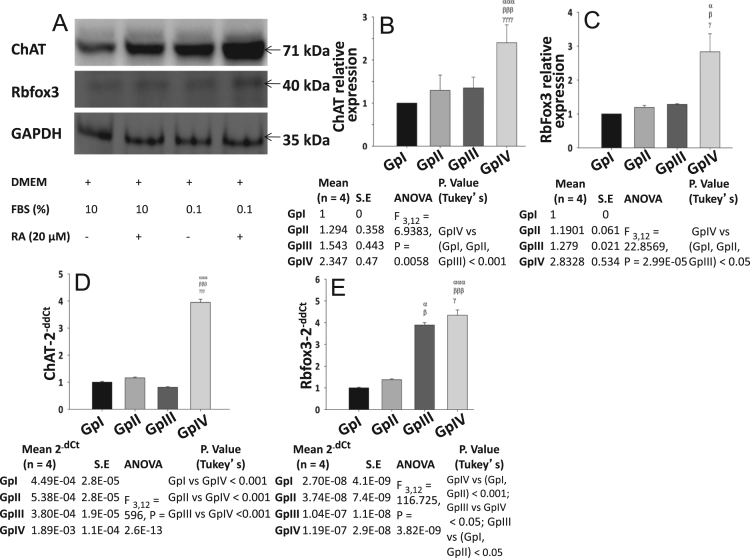
Protein and mRNA expression data for the neuronal marker of differentiation using various serum concentrations in the presence/absence of retinoic acid. A; Represents western blot of ChAT, Rbfox3, GAPDH. B, C; Relative quantification of ChAT and Rbfox3 w.r.t. GAPDH as the reference control. D, E; Real-time PCR data for ChAT and Rbfox3, analyzed by the 2^-ddCt^ method using SDHA as the reference gene. Values are mean ± S.E. *P* ≤ 0.05 (*α*, *β*, *γ*), *P* ≤ 0.01 (*αα*, *ββ*, *γγ*), *P* ≤ 0.001 (*ααα*, *βββ*, *γγγ*) were considered to be statistically significant. *α*, Compared to GpI; *β*, Compared to GpII; *γ*, Compared to GpIII. Additionally, mean values, S.E., ANOVA, and Tukey׳s multiple comparisons statistics are shown in tabular form below their respective graphs.

Neuro-2a cells (also known as N2a) were subjected to various nutrient culture conditions, namely DMEM + 10% FBS (GpI), DMEM + 10% FBS + 20 μM Retinoic acid (GpII), DMEM + 0.1% FBS (GpIII), DMEM + 0.1% FBS + 20 μM Retinoic acid (GpIV), to observe the effect on cellular differentiation. Phase contrast microscopy (Fig. 1-A–D) was used to visualize the morphological changes (neurite extension) induced by various treatment conditions. Extensive increase (*P* < 0.001) in the neurites length (Fig. 1E) and branching pattern (Fig. 1F) was seen in GpIV at 24 h, whereas GpIII did show moderate neurite extension. CFSE assay was performed to assess the rate of cellular proliferation and data for the same are plotted as bar graphs (Fig. 2-E, F). The difference in the mean fluorescence intensity between GpIV and other groups was observed, which indicated the low rate of proliferation in GpIV. Additionally, it has been observed in previous reports that RA induces cell cycle arrest at the G_0_/G_1_ phase [1]. In order to delineate this type of response, cell cycle analysis was performed (Fig. 2-A–D) which indicated an increase in the number of cells arrested at G_0_/G_1_ phase in GpIII (53.25 ± 0.25%) and GpIV (67.76 ± 2.68%). Furthermore, mRNA and protein expressions of Rbfox3 (neuronal differentiation marker) and ChAT (cholinergic neuronal marker) were assessed using real-time PCR (Fig. 3-D, E) and western blot analysis (Fig. 3-A–C). The increased expression (~ 4 fold) of both the markers upon differentiation was observed in GpIV.

## 2 Experimental design, materials and methods

### 2.1 Neuro-2a cell culture and differentiation

Neuro-2a cells were procured from NCCS Pune, India and maintained in DMEM (Sigma, D7777) supplemented with 10% FBS (Thermo Fisher Scientific, 10270106) at 37 °C, 5% CO_2_ conditions. For differentiation, the cells were plated at a density of 2 × 10^4^ cells/cm^2^ and maintained in the standard growth media for 24 h. Next day, the standard growth media were replaced with the differentiation media of various compositions (Table 1) in their respective wells and incubation was continued for the next 24 h. Retinoic acid (R2625) was procured from Sigma-Aldrich.

**Table 1:** Compositions of differentiation media.

Label	Culture media	Fetal bovine serum (FBS)	Retinoic acid (μM)
GpI	DMEM	10%	0
GpII	DMEM	10%	20
GpIII	DMEM	0.10%	0
GpIV	DMEM	0.10%	20

### 2.2 Neurite length measurement

Neuro-2a cells were grown in 6-well multi-dishes (Thermo Fischer Scientific, 140675) and differentiated as per above method. Images were captured with the Nikon Eclipse TS100 at 20× magnification using VEZU US300. Neurite length was measured using ImageJ-Simple Neurite Tracer plugin [2]. Briefly, Images were first converted to 8-bit luminance format for analysis by the plugin. For defining neurite׳s path, a manual tracking was done from the junction of the cell body and neurite up to the neurite tip. Only neurites > 1 μM in length were taken for final analysis. Branching patterning was further assessed using the Sholl analysis function in the same plugin. The neurite׳s path was manually defined, followed by assigning soma as a center of analysis. Using plugin parameters (Standard axis, Step radius = 0 μM, Enclosing radius cut off = 1, Sholl methods = Linear, Polynomial = Best fitting degree, Normalized profile = Best fitting), Sholl analysis was performed. Maximum intersections score thus obtained reflect the path having the highest level of branches and was taken as a measure for showing branching level (Branching level = 1 − Intersections score = 1). Moreover, cell density (cells/475 μm^2^) was calculated using Analyze Particles function׳s parameters (Threshold: Lower = 123; Upper = 255; Size = 0.2–4 μm^2^; Circularity = 0–1) in ImageJ.

### 2.3 Cell cycle analysis

Cell cycle analysis was performed as per Lisa et al. [3]. Briefly, upon completion of 24 h of differentiation, 1 × 10^6^cells were harvested by trypsinization (0.25%), resuspended in 1 ml of ice-cold PBS (137 mM NaCl, 2.7 mM KCl, 10 mM Na_2_HPO_4_, 1.8 mM KH_2_PO_4_). 100% ice-cold ethanol (Merck, 100983) was added dropwise, and the mixture was incubated at 4 °C for 20 min. Cells were then washed with ice-cold PBS and incubated in the staining solution (30 µg/mL, Propidium iodide, Sigma-Aldrich, P4170; 100 µg/mL, RNaseA, Thermo Fisher Scientific, EN0531). Scanning of samples was done using FACS Calibur™ (Detector settings: - FSC: Voltage = E00, Amp gain = 1.45, Lin; SSC: Voltage = 346, Amp gain = 1, Lin; FL-2: Voltage = 564, Lin) and data were analyzed using BD CellQuest™ Pro.

### 2.4 CFSE assay

Before starting the process of neuro-2a cellular differentiation, cells were processed for CFSE as per manufacturer protocol (CellTrace™, Thermofisher, C34554). Briefly, cells were incubated at 37 °C in a staining mixture of CFSE for 20 min, followed by further incubation in complete media for 5 min. Subsequently, complete media were replaced with the differentiation media. After completion of differentiation protocol, cells were trypsinized and scanned with FACS Calibur^TM^ (Detector settings: - FSC: Voltage = E00, Amp gain = 1.45, Lin; SSC: Voltage = 346, Amp gain = 1, Lin; FL-1: Voltage = 350, Log).

### 2.5 RNA extraction, cDNA synthesis and real-time PCR

RNA was extracted using Trizol^R^ method (Thermo Fisher Scientific, 15596026). The integrity of RNA was checked using NanoDrop 260/280 ratio (1.8–2.2) and RNA gel electrophoresis [4]. Reverse transcription was done using Maxima First Strand cDNA Synthesis Kit (Thermo Fischer Scientific, K1671) as per manufacturer protocol. Further, ~ 30 ng cDNA was used as the template for real-time PCR using Primer3 designed primer pairs, i.e., ChAT, FP-5’-ATGAACGCCTGCCTCCAATCGG-3’, RP-5’-CAGATGCAGCGCTCGATCATG-3’; Rbfox3, FP-5’-CTCCAACCCGGCCTCTC-3’, RP-5’-GCACTAGGTTCTCACAGGCA-3’; SDHA, FP-5’-TGGAAGATCTCTGCGATATGACAC-3’, RP-5’-TTCGGTGTATGGACCCATCTTCTA-3’ as per Maxima SYBR Green/ROX qPCR Master Mix (Thermo Fischer Scientific, K0221). Biorad CFX96 Real-Time PCR was used for PCR amplification and detection. The data were analyzed with the 2^-ddCt^ method.

### 2.6 Protein extraction and western blotting

Proteins were extracted in RIPA Buffer (150 mM NaCl, 1.0% IGEPAL^®^CA-630, 0.5% Sodium deoxycholate, 0.1% SDS, 50 mM Tris, pH 8.0; Sigma). ~ 50 μg proteins were electrophoresed on 12% PAGE and electroblotted (100 V, 1 h) onto the nitrocellulose membrane. Blots were blocked with 5% BSA, 1 h at room temperature and then incubated with primary antibodies, i.e., ChAT (1:500, Santa Cruz Biotechnology, sc-55557), Rbfox3 (1:500, Santa Cruz Biotechnology, sc-2469557) respectively for overnight at 4 °C. Samples were further incubated with anti-mouse IgG-HRP (1:5000, Santa Cruz Biotechnology) and anti-rabbit IgG-HRP (1:5000, Santa Cruz Biotechnology) respectively for 1 hr. Blots were developed with Clarity^TM^ Western ECL Substrate (Biorad, 170–5060) and imaged with Fuji-LAS4000 luminescent image system (GE Healthcare).

### 2.7 Statistical analysis

Values are presented as the mean±S.E. Statistical analysis was done with SigmaPlot 11.0 with one way ANOVA and post hoc analysis using Tukey׳s multiple comparison test. *P* ≤ 0.05, *P* ≤ 0.01, *P* ≤ 0.001 were considered to be statistically significant.
